# Oxygen-Sensing Protein Cysteamine Dioxygenase from Mandarin Fish Involved in the Arg/N-Degron Pathway and *Siniperca chuatsi* Rhabdovirus Infection

**DOI:** 10.3390/v15081644

**Published:** 2023-07-28

**Authors:** Wenhui Liu, Jian He, Zhimin Li, Shaoping Weng, Changjun Guo, Jianguo He

**Affiliations:** 1 State Key Laboratory for Biocontrol, Guangdong Provincial Key Laboratory of Marine Resources and Coastal Engineering, School of Marine Sciences, Sun Yat-sen University, Guangzhou 510275, China; liuwh6@mail2.sysu.edu.cn (W.L.); hejian35@mail.sysu.edu.cn (J.H.); lizhm6@mail2.sysu.edu.cn (Z.L.); lsshjg@mail.sysu.edu.cn (J.H.); 2 Guangdong Provincial Observation and Research Station for Marine Ranching of the Lingdingyang Bay, Southern Marine Science and Engineering Guangdong Laboratory (Zhuhai), Zhuhai 519082, China; 3 Guangdong Province Key Laboratory for Aquatic Economic Animals, School of life Sciences, Sun Yat-sen University, Guangzhou 510275, China; lsswsp@mail.sysu.edu.cn

**Keywords:** hypoxia, ADO, SCRV, *Siniperca chuatsi*

## Abstract

Mammalia cysteamine (2-aminoethanethiol) dioxygenase (ADO) controls the stability of the regulator of G protein signaling 4 (RGS4) through the Cys branch of the Arg/N-degron pathway, thereby affecting the response of the body to hypoxia. However, the oxygen-sensing function of ADO remains unknown in teleost fish. Mandarin fish (*Siniperca chuatsi*) is one of the most important freshwater economic fishes in China. As the scale of the rearing density continues to increase, hypoxia has become an important factor threatening the growth of mandarin fish. Herein, the molecular characterization, the oxygen-sensing enzyme function, and the role in virus infection of ADO from mandarin fish (*sc*ADO) were explored. Bioinformation analysis results showed that *sc*ADO had all the molecular foundations for achieving thiol dioxygenase function: three histidine residues coordinated with Fe(II), PCO/ADO domain, and a “jelly roll” β-barrel structure. The expression pattern analysis showed that *scAdo* was highly expressed in the immune-related tissues, liver, and kidneys and responded to hypoxia on the expression level. Protein degradation experiment results revealed that *sc*ADO could lead to the degradation of RGS4 protein through the Cys branch of the Arg/N-degron pathway. Furthermore, the expression levels of *sc*ADO responded to fish virus infection. *sc*ADO could significantly promote the replication of *Siniperca chuatsi* rhabdovirus, and this was associated with its thiol dioxygenase activity. These findings not only demonstrate *sc*ADO as an oxygen-sensing protein in teleost fish, but are also of considerable importance for clarifying the contribution of the mechanism of hypoxia to the outbreaks of fish viruses.

## 1. Introduction

Eukaryotic organisms have two kinds of oxygen-sensing mechanisms. The first mechanism is based on Fe(II)/2-oxoglutarate-dependent dioxygenases, represented by prolyl hydroxylases (PHDs) and factor-inhibiting hypoxia-inducible factor 1 involved in the hypoxia-inducible factor (HIF) signaling pathway [1,2,3,4], which has been extensively studied. The other mechanism is based on thiol dioxygenases, plant cysteine oxidases (PCOs) [5,6], and cysteamine (2-aminoethanethiol) dioxygenase (ADO) [7,8], regulating the response to changes in oxygen conditions through the cysteine (Cys) branch of the Arg/N-degron pathway [9,10,11]. They can specifically recognize the Cys residue at the N-terminal of proteins and perform dioxygenation modification on the thiol group of Cys. Arginyl-transferase 1 (ATE1) adds arginine to the oxidized Cys, leading to its recognition by E3 ubiquitin ligases and degradation by proteasomes [12,13]. The oxygen-sensing enzyme function of ADO in humans was not confirmed until recent years. *Homo sapiens* ADO (*h*ADO) catalyzes the oxidation of the N-terminal Cys of the regulator of G protein signaling 4 (RGS4) proteins, leading to their degradation under normoxic conditions [8]. The activity of *h*ADO is limited under hypoxic conditions, and the stable presence of RGS4 proteins can enhance the hydrolysis of GTP coupled with Gα, leading to the weakening of G protein signal, which then results in the weakening of Ca^2+^ release and the reduction in the MAPK phosphorylation level. However, current research on ADO in other species is even rare.

Mandarin fish (*Siniperca chuatsi*) is an important economically cultured fish, with a requirement for a dissolved oxygen concentration of above 5 mg/L in water, and its growth and development are affected when this concentration is below 3 mg/L. Hypoxia often occurs in aquatic environments in the case of intensive aquaculture [14,15,16], which frequently contributes to the outbreak of infectious diseases and severe economic loss [17,18]. The three most harmful viruses are as follows: infectious spleen and kidney necrosis virus (ISKNV) [19], Mandarin fish ranavirus (MRV) [20], and *Siniperca chuatsi* rhabdovirus (SCRV) [21,22]. Previous study has shown that hypoxia could induce the expression of viral genes through viral hypoxia response elements (HREs), which promoted the replication of ISKNV [23], suggesting the involvement of hypoxic aquatic environments in the outbreak of viral infectious diseases for mandarin fish. Therefore, conducting a profound study on the oxygen-sensing mechanisms in mandarin fish is of considerable importance for the prevention and control of viral diseases.

The molecular characterization, the oxygen-sensing enzyme function, and the role in the virus infection of ADO from mandarin fish (*sc*ADO) were explored in the present study. This study is crucial for understanding the role of *sc*ADO in the outbreak of aquatic virus diseases.

## 2. Materials and Methods

### 2.1. Cell, Fish, and Hypoxic Exposure

Mandarin fish fry (MFF-1) cell line was constructed and maintained in the laboratory and was cultured in Dulbecco’s modified Eagle’s medium (Gibco, Waltham, MA, USA) containing 10% fetal bovine serum (FBS; Gibco) at 27 °C and 5% CO_2_ [24]. Fathead minnow (FHM) cell line was purchased from the American Type Culture Collection and was also maintained in the laboratory; it was cultured in M199 medium containing 10% FBS at 27 °C and 5% CO_2_ [25]. Ten healthy mandarin fish (body weight of 75–100 g) were purchased from a farm in Guangdong province. They were bred in a laboratory recirculating fresh water system for two weeks to facilitate acclimatization, and the water temperature was maintained at 27 °C. Hypoxic incubations were conducted within the hypoxic cell incubator set at 1–3% O_2_, 5% CO_2_, balance N_2_.

### 2.2. Virus and Infection

The ISKNV (strain OP896201.1), MRV (strain MG941005.3), and SCRV (strain NC_008514.1) were separated from the diseased mandarin fish and stored in the laboratory. The virus suspension was diluted with culture medium (containing 10% FBS) in the following proportion: multiplicity of infection (MOI) = 1. Cells were cultured for more than 24 h and then exposed to the culture medium containing virus. The virus was removed after incubation for 2–4 h, and the new culture medium was added for further culture.

### 2.3. Antibodies and Reagents

Antibodies specific for MYC-tag and β-actin were obtained from Proteintech (Chicago, IL, USA), and those specific for HA-tag were obtained from Abcam (Cambridge, MA, USA). Alexa Fluor 594-labeled goat anti-rabbit IgG and Hoechst 33342 were obtained from Thermo Fisher Scientific (Waltham, MA, USA). The cycloheximide (CHX), MG132, and tannic acid were purchased from MedChem Express (Monmouth Junction, NJ, USA), and 2,2-DIP was purchased from Solarbio (Beijing, China).

### 2.4. Molecular Cloning of Mandarin Fish ADO and RGS4 cDNAs

The *sc*ADO and *sc*RGS4 sequences were obtained from the transcriptome data of *Siniperca chuatsi* (GCA_027580155.1), and the primers used for cloning are shown in Table 1. The PCR amplification was performed as previously described [26].

### 2.5. Sequence Analysis

The multiple sequence alignments were generated using the ClustalW program (http://www.genome.jp/tools-bin/clustalw, accessed on 1 March 2023). The predicted amino acid sequence of *sc*ADO was analyzed using the Simple Modular Architecture Research Tool (SMART) program (http://smart.embl-heidelberg.de, accessed on 1 March 2023). The three-dimensional protein structure was predicted by applying the homology modeling technique in SWISS-MODEL (http://swissmodel.expasy.org, accessed on 1 March 2023). The phylogenetic tree of ADO sequences was constructed in accordance with the alignment of amino acid sequences through the neighbor-joining method using the Molecular Evolutionary Genetics Analysis (MEGA) v7.0 program, with 1000 bootstrap replicates.

### 2.6. Tissue Distribution Analysis

Different tissue samples, including liver, blood, fin, intestine, brain, gonad, heart, gill, pronephros, mesonephros, metanephros, and spleen, were separated from the healthy mandarin fish, and total RNAs were extracted using TRIzol reagent (Thermo Fisher Scientific, USA) according to the manufacturer’s protocol as previously described [27]. An amount of 500 ng of each RNA sample was taken to synthesize cDNA immediately after extraction.

### 2.7. Quantitative Reverse Transcription PCR (RT–qPCR)

The RT-qPCR was performed with SYBR^®^ premix ExTaq^TM^ (Takara, Tokyo, Japan) on a LightCycler 480 instrument (Roche Diagnostics, Rotkreuz, Switzerland). Primers for RT–qPCR were designed using Primer 6.0 software and are shown in Table 2. The RT-qPCR conditions were as previously described [28].

### 2.8. Cell Transfection

Following standard methods, transient transfection of plasmids was conducted with MIK X Transfection Reagent (MIK, Guangzhou, China) according to the manufacturer’s instructions.

### 2.9. Immunofluorescence Assay (IFA)

The Endo-free *sc*ADO-MYC and pCMV-MYC plasmids were transfected into FHM cells using MIK X transfection reagent. The IFA was performed as previously described after 48 h [29].

### 2.10. Western Blot

Cells were harvested and lysed. The lysates were mixed with 5× loading buffer, boiled for 10 min to prepare protein samples, and subjected to SDS-PAGE for separation. The proteins were subsequently transferred onto polyvinylidene fluoride membranes. The membranes were blocked in 5% skim milk in TBST buffer at room temperature for 1 h and then washed three times with TBST for 5 min each time. Subsequently, the membranes were incubated with the appropriate primary and secondary antibodies at room temperature. Protein bands were visualized using a High-sig Chemiluminescence (ECL) Western Blotting Substrate kit after extensive washing (Tanon, Shanghai, China).

### 2.11. TCID_50_ Assay

The TCID_50_ value was calculated using Spearman–Karber method as previously described [30,31]. Briefly, MFF-1 cells were seeded in 96-well cell culture plates and cell density exceeded 80%. The original samples were diluted to 10^−1^–10^−10^ using DMEM. Each concentration gradient of the virus was added into eight wells, and 100 μL of DMEM without virus was also placed in eight wells as negative control. Subsequently, the 96-well cell culture plates were incubated at 27 °C and 5% CO_2_. The number of positive and negative wells were observed and recorded daily, generally lasting for approximately five days.

## 3. Results

### 3.1. Bioinformatics Analysis of scADO

**Figure 1:**
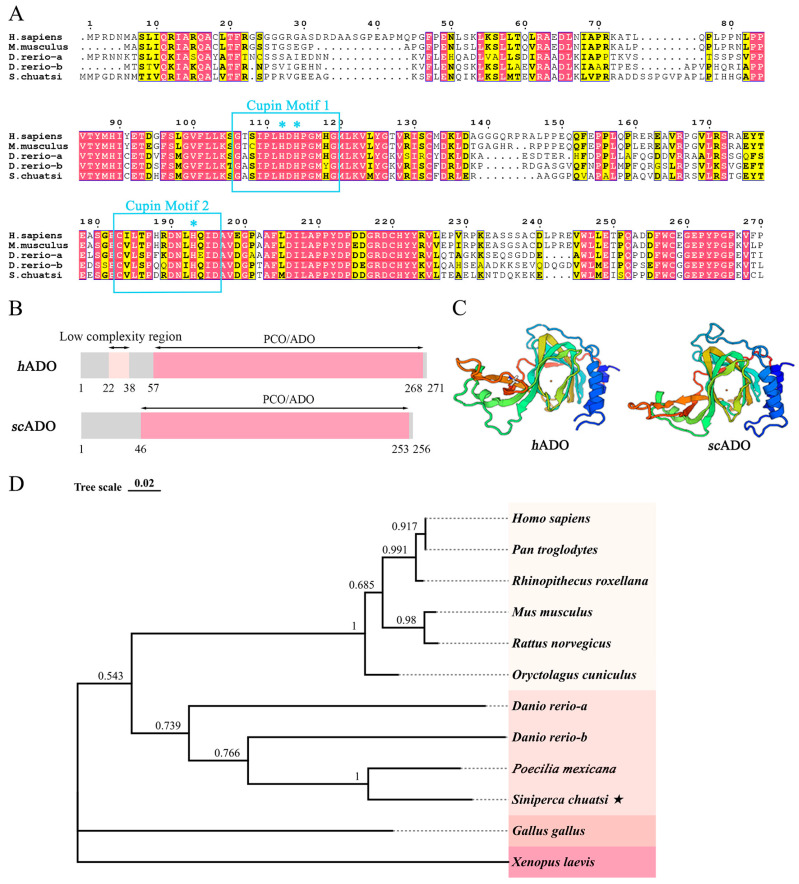
Sequence analysis of *sc*ADO. (**A**) Alignment of amino acid sequence. * Represents the essential His residues contributed to the coupling with Fe(II). (**B**) Protein domain predicted by SMART. (**C**) Crystal structure predicted by SWISS-MODEL. The rainbow color begins with blue at the N-terminus to red at the C-terminus. (**D**) Phylogenetic analysis was conducted in MEGA v7.0 using the neighbor-joining method. The number next to the branches show the percentage of associated taxa clustered together in the bootstrap test (1000 replicates). Scale bar is 0.02. ★ marked mandarin fish (*Siniperca chuatsi*).

Through sequence alignment, a gene highly homologous to *hAdo* was selected from the transcriptome data of *Siniperca chuatsi* and was cloned, and a 768 bp full length sequence was obtained. Biological analysis was conducted on its sequence to investigate the characterization of *scAdo*. The alignment results of amino acid sequences reveal that *sc*ADO shares a common architecture with *h*ADO and comprise two cupin metalloenzyme motifs of cupin superfamily proteins, namely Cupin Motif 1 (GX_5_HXHX_3,4_EX_6_G) and Cupin Motif 2 (GX_5_PXGX_2_HX_3_N) [32] (Figure 1A). The three His residues in the two motifs have been proven to be ligands for ADO to bind to Fe(II), which is a necessary prerequisite for its thiol dioxygenase activity.

The SMART domain prediction analysis result showed that *sc*ADO protein had a PCO/ADO domain (Figure 1B), which was a molecular basis for exerting the function of thiol dioxygenase. The protein structure predicted by the SWISS-MODEL also indicated that the crystal structure of *sc*ADO was consistent with that of *h*ADO, both comprising a “jelly roll” β-barrel that supported a catalytic center [33] (Figure 1C). The phylogenetic analysis result showed that *scAdo* was clustered with other species and had high homology with fish *Ado* genes (Figure 1D). These results suggested that the ADO of mandarin fish was cloned and identified.

### 3.2. Expression Patterns and Subcellular Localization of scAdo

**Figure 2:**
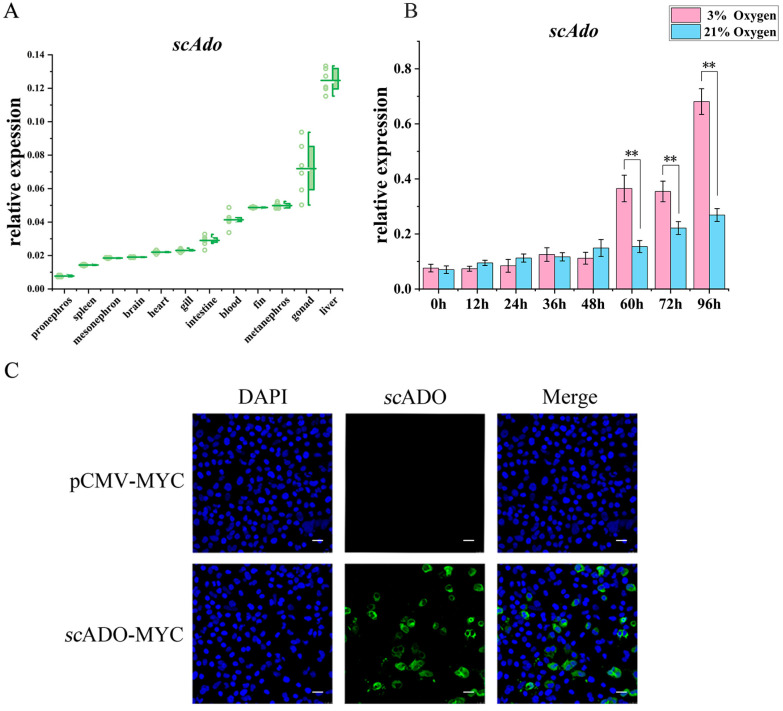
Expression patterns and subcellular localization of *sc*ADO. (**A**) Expression level of *scAdo* in tissues of mandarin fish. (**B**) Expression level of *scAdo* at different oxygen concentrations in MFF-1 cells. (**C**) The subcellular localization of *sc*ADO, which has a scale bar of 10 μm, *sc*ADO labeled by green fluorescence and cell nucleus labeled by blue fluorescence. The data of qRT–PCR are shown as mean ± SD (*n* = 3), and asterisks represent statistical differences, ** *p* < 0.01.

The RT-qPCR was conducted to investigate the distribution of *scAdo* in various tissues of mandarin fish. Figure 2A shows that the relative expression of *scAdo* mRNA was the highest in the liver, followed by the gonad. A high level of *scAdo* mRNA expression was also observed in the kidneys, fin, blood, and intestine, while the expression was relatively low in the gill, heart, brain, and spleen. Among them, the liver and kidneys were important immune tissues in fish, and it is speculated that the high expression of *scAdo* in immune-related tissues might involve in the immune response of mandarin fish.

The expression pattern of *scAdo* was explored under hypoxia due to *h*ADO, an oxygen-sensing enzyme that can respond to changes in oxygen levels [7]. The results showed that the relative expression of *scAdo* mRNA significantly increased after culturing cells at 3% O_2_ for 60 h compared to that in the control group with 21% O_2_, indicating that hypoxia could significantly induce the expression of *scAdo* (Figure 2B). Moreover, the IFA result showed that *sc*ADO mainly existed in the cytoplasm (Figure 2C), indicating its possible role in the cytoplasm.

### 3.3. scADO Could Regulate the Stability of hRGS4

**Figure 3:**
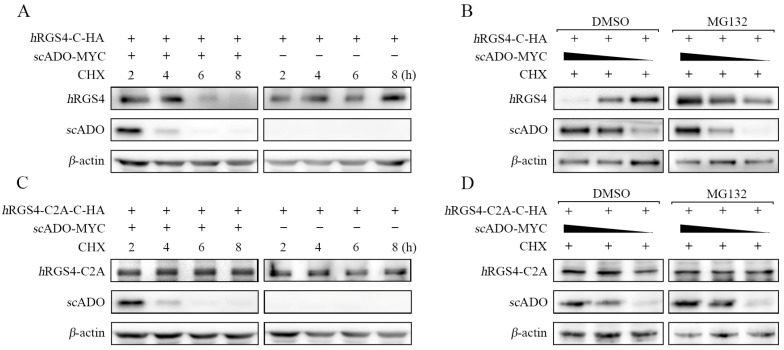
Regulation of the stability of *h*RGS4 and *h*RGS4-C2A by *sc*ADO. (**A**) *h*RGS4 protein levels after co-expression with empty vector or *sc*ADO in FHM cells were treated with CHX. (**B**) *h*RGS4 protein levels after co-expression with different dosages of *sc*ADO in FHM cells were treated with CHX and DMSO or MG132. (**C**) Protein levels of *h*RGS4-C2A mutant after co-expression with empty vector or *sc*ADO in FHM cells were treated with CHX. (**D**) Protein levels of *h*RGS4-C2A mutant after co-expression with different dosages of *sc*ADO in FHM cells were treated with CHX and DMSO or MG132. All concentrations of CHX and MG132 used were 20 mM unless otherwise indicated.

This study also explored whether *sc*ADO has similar functions to *h*ADO, leading to the specific degradation of RGS4 [8,16]. Thus, the gene sequence of *h*RGS4 was cloned and constructed into the pCMV-C-HA vector to ensure that the N-terminal Cys residue of the *h*RGS4 protein could be exposed for recognition. Cells were co-transfected with the *h*RGS4-C-HA plasmid and *sc*ADO-MYC/pCMV-MYC and then treated with cycloheximide (CHX) to inhibit intracellular protein synthesis after 16 h of transfection. The results showed that the protein level of *h*RGS4 continuously decreased with the prolongation of CHX treatment time in the presence of *sc*ADO and was almost undetectable by 8 h (Figure 3A). This phenomenon suggests that *sc*ADO might lead to the degradation of *h*RGS4, which also degraded during this process. Cells were co-transfected with the *h*RGS4-C-HA plasmid with different doses of *sc*ADO-MYC plasmid for 16 h and were then treated with CHX and MG132 to further validate this result. As shown in Figure 3B, the protein level of *h*RGS4 increased with a decrease in *sc*ADO in a dose-dependent effect when MG132 was not used. This effect disappeared after MG132 treatment. These results suggested that *sc*ADO could lead to the degradation of *h*RGS4, which might be achieved through the ubiquitin proteasome pathway.

Previous research results revealed that the regulation of *h*RGS4 stability by *h*ADO depends on the Cys in the second position of its amino acid sequence. An *h*RGS4-C2A-C-HA mutant was constructed to further explore the mechanism consistency of *sc*ADO leading to *h*RGS4 degradation with *h*ADO. Cells were co-transfected with *h*RGS4-C2A-C-HA mutant with the *sc*ADO-MYC or pCMV-MYC plasmid for 16 h and were then treated with CHX. As shown in Figure 3C, the presence or absence of *sc*ADO did not affect the protein level of *h*RGS4-C2A. Similarly, in cells treated with CHX and MG132 (Figure 3D), the dose-dependent degradation effect of *sc*ADO on *h*RGS4 did not appear on *h*RGS4-C2A regardless of MG132 treatment. These results were sufficient to prove that the degradation effect of *sc*ADO on *h*RGS4 was based on the Cys in the second position of the *h*RGS4 amino acid sequence, which was consistent with the *h*ADO regulation mechanism of *h*RGS4 stability. The above observations suggested that *sc*ADO might share a similar role to *h*ADO in regulating the stability of *h*RGS4. Meanwhile, during the degradation of *h*RGS4, *sc*ADO also degraded, but the specific mechanism was still unknown. In order to determine whether these two proteins degraded together after their interaction, or *sc*ADO itself had a degradation mechanism, further research is needed.

### 3.4. scADO Regulated the Stability of scRGS4 through the Cys Branch of the Arg/N-Degron Pathway

**Figure 4:**
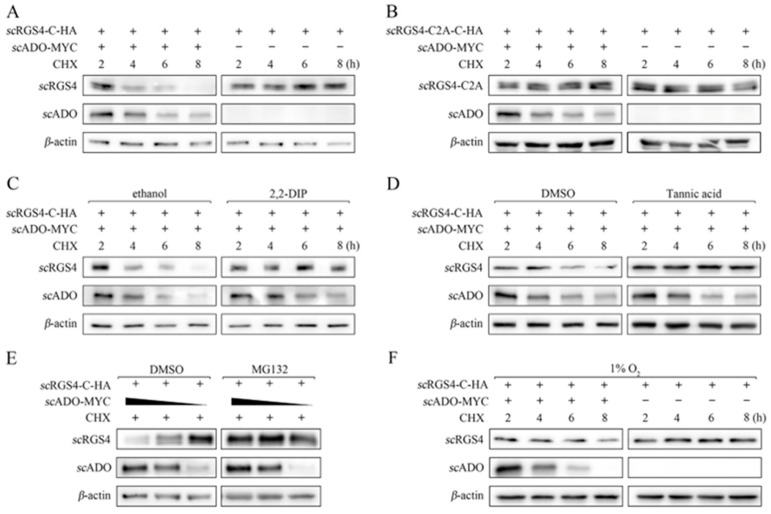
Regulation of the stability of *sc*RGS4 and *sc*RGS4-C2A by *sc*ADO. (**A**) *sc*RGS4 protein levels after co-expression with empty vector or *sc*ADO in FHM cells were treated with CHX. (**B**) Protein levels of *sc*RGS4-C2A mutant after co-expression with empty vector or *sc*ADO in FHM cells were treated with CHX. (**C**) *sc*RGS4 protein levels after co-expression with *sc*ADO in FHM cells treated with CHX and ethanol or 2,2-DIP. (**D**) *sc*RGS4 protein levels after co-expression with *sc*ADO in FHM cells were treated with CHX and DMSO or tannic acid. (**E**) *sc*RGS4 protein levels after co-expression with different dosages of *sc*ADO in FHM cells were treated with CHX and DMSO or MG132. (**F**) *sc*RGS4 protein levels after co-expression with empty vector or *sc*ADO in FHM cells were treated with CHX and exposed to 1% O_2_. All concentrations of 2,2-DIP and tannic acid used were 100 μM unless otherwise indicated.

The *scRgs4* gene and its C2A mutant were cloned into the pCMV-C-HA vector to verify whether *sc*ADO could perform this function in mandarin fish. Cells were co-transfected with *sc*RGS4-HA or its mutant as well was the *sc*ADO-MYC or pCMV-MYC plasmid for 16 h and were then treated with CHX. As shown in Figure 4A, the degradation effect of *sc*ADO on *sc*RGS4 was consistent with its degradation effect on *h*RGS4, and *sc*RGS4 protein was almost undetectable after 8 h of CHX treatment. Meanwhile, the retained protein level of *sc*RGS4-C2A in cells was co-transfected with *sc*ADO-MYC and *sc*RGS4-C2A (Figure 4B), indicating that the degradation of *sc*RGS4 by *sc*ADO must be based on its N-terminal Cys residue. The specific inhibitors of ADO (2,2-DIP) and ATE1 (tannic acid) were used to further investigate the *sc*ADO degradation of *sc*RGS4 through the Cys branch of the Arg/N-degron pathway. The *sc*RGS4 was no longer degraded after cells were treated with 2,2-DIP, while the overexpression of *sc*ADO avoided the degradation of *sc*RGS4 under the treatment with tannic acid (Figure 4C). These results suggested that the degradation of *sc*RGS4 was achieved through the thiol dioxygenase activity of *sc*ADO, and the arginylation of ATE1 was an essential step in the degradation of *sc*RGS4.

Subsequently, cells were co-transfected with *sc*ADO-MYC and *sc*RGS4-HA and were then treated with MG132 to investigate the degradation of *sc*RGS4 by *sc*ADO via the proteasome pathway. Figure 4E shows that *sc*ADO no longer had an impact on the protein level of *sc*RGS4 when cells were treated with MG132, whereas the *sc*ADO led to the degradation of *sc*RGS4 with a dose-dependent manner in the control cells. This observation proved that *sc*RGS4 was degraded via the proteasome pathway. Furthermore, the role of oxygen in the entire process was explored. The overexpression of *sc*ADO still led to the degradation of *sc*RGS4 at an extremely low oxygen content of 1% O_2_. However, the degradation rate was significantly reduced compared to normal oxygen conditions (Figure 4F), indicating that the thiol dioxygenase activity of *sc*ADO was influenced by oxygen content, yet also failed to tolerate a certain degree of hypoxia. The above results suggested that *sc*ADO was an oxygen-sensing enzyme and regulated the stability of *sc*RGS4 through the Cys branch of the Arg/N-degron pathway.

### 3.5. scADO Promoted SCRV Replication

**Figure 5:**
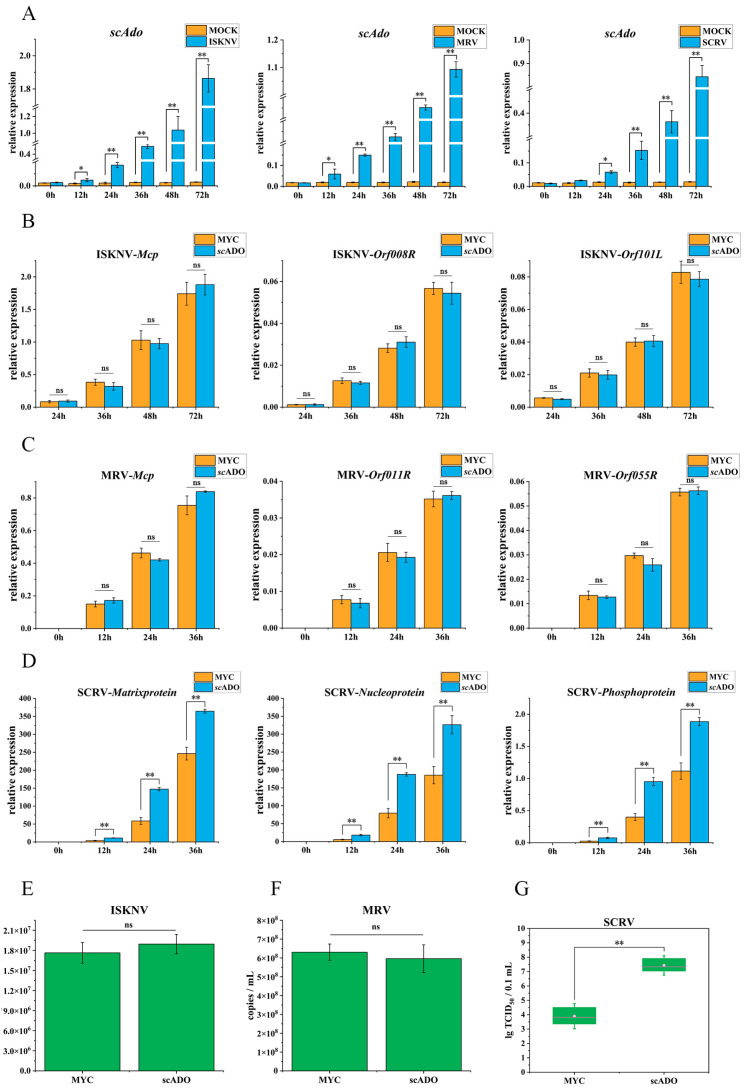
Impact of *sc*ADO on virus replication. (**A**) The relative expression level of *scAdo* after ISKNV, MRV, and SCRV infections in MFF-1 cells. (**B**) The relative expression level of ISKNV genes. (**C**) The relative expression level of MRV genes. (**D**) The relative expression level of SCRV genes. (**E**) ISKNV viral load of cells transfected with *sc*ADO or not. (**F**) MRV viral load of cells transfected with *sc*ADO or not. (**G**) SCRV TCID_50_ value of cells transfected with *sc*ADO or not. The data are shown as mean ± SD (*n* = 3), where asterisks represent statistical differences (* *p* < 0.05, ** *p* < 0.01 and ns represents not significant).

The expression levels of *scAdo*, which were responses to ISKNV, MRV, or SCRV infection, were analyzed to further explore the involvement of *sc*ADO in the immune process. As shown in Figure 5A, the expression of *scAdo* showed significant upregulation after infection with the three viruses. These results indicated that virus infection could induce the expression of *scAdo*, and *sc*ADO might play a role in the infection process of virus. The effect of *sc*ADO separately overexpressed on the replications of three viruses was analyzed to further investigate the role of *sc*ADO in virus infection. The relative expression levels of three commonly used genes in virus detection were detected to characterize the replication of the three viruses: *Mcp* encoding major capsid protein, immediate early gene *Orf008R*, and *Orf101L* encoding immunogenic protein for ISKNV; *Mcp*, immediate early gene *Orf011R*, and *Orf055R* encoding DNA polymerase for MRV; *matrixprotein*, *nucleoprotein*, and *phosphoprotein* for SCRV. The *sc*ADO did not have any effect on the expression levels of ISKNV and MRV (Figure 5B,C) viral genes, whereas the expression levels of SCRV viral genes were significantly promoted (Figure 5D). Similarly, *sc*ADO had no effect on the DNA replication of ISKNV and MRV (Figure 5E,F). The viral loads were determined in cells transfected with *sc*ADO-MYC using TCID_50_ assay to verify the role of *sc*ADO in SCRV infection. As shown in Figure 5G, the viral load in cells overexpressing *sc*ADO-MYC was significantly higher than that in cells transfected with empty vector. The above results suggested that *sc*ADO played an important role in SCRV infection and could promote the replication of SCRV.

**Figure 6:**
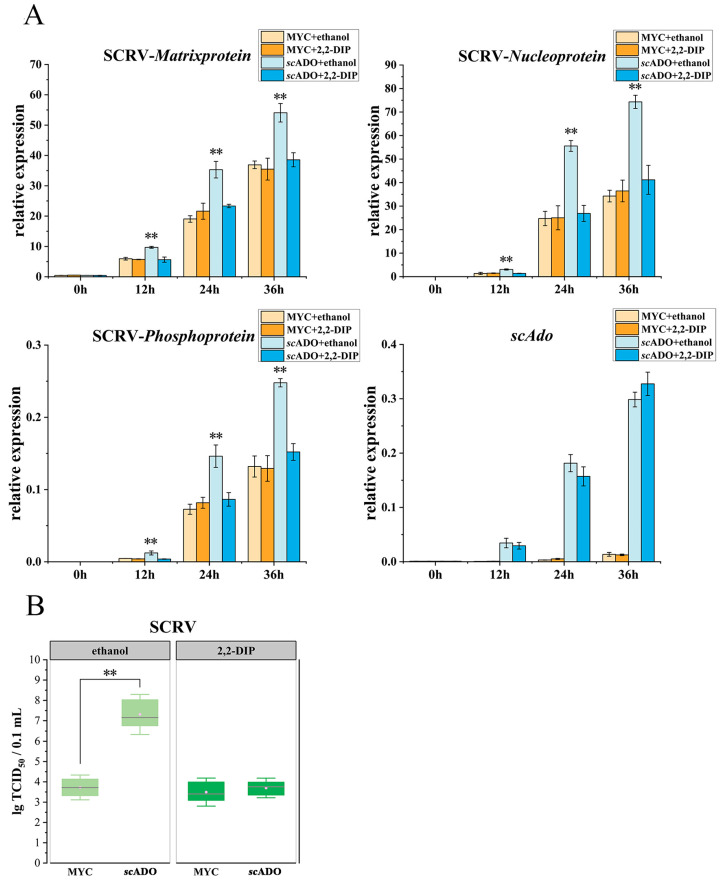
2,2-DIP inhibited the role of *sc*ADO in promoting SCRV replication. (**A**) The relative expression level of SCRV genes in MFF-1 cells. (**B**) TCID_50_ value of corresponding cells. The data are shown as mean ± SD (*n* = 3), and asterisks represent statistical differences, ** *p* < 0.01. All concentrations of 2,2-DIP and ethanol used were 100 μM unless otherwise indicated.

Cells were transfected with *sc*ADO-MYC/pCMV-MYC and then treated with 100 μM 2,2-DIP or ethanol to further investigate whether the promoting effect of *sc*ADO on SCRV replication was related to its thiol dioxygenase activity. As shown in Figure 6A, 2,2-DIP did not affect the replication of SCRV (MYC+2,2-DIP group) compared to the control cells (MYC + ethanol group). *sc*ADO significantly promoted the expression of SCRV genes when cells were not treated with 2,2-DIP (*sc*ADO + ethanol group), but the promoting effect of *sc*ADO disappeared under the treatment of 2,2-DIP (*sc*ADO+2,2-DIP group). The results of TCID_50_ assay also demonstrated that the viral load of SCRV decreased after treatment with 2,2-DIP in cells overexpressing *sc*ADO, which was essentially the same as the viral load level in control groups (Figure 6B). These results suggested that 2,2-DIP could inhibit the promotion of SCRV replication by *sc*ADO, indicating that the *sc*ADO promotion of SCRV replication was associated with its thiol dioxygenase activity.

## 4. Discussion

In recent years, it has been confirmed that *h*ADO regulates the stability of RGS4 through the Cys branch of the Arg/N-degron pathway, thereby affecting the response of the body to hypoxia [8]. However, the oxygen-sensing function of ADO remains unknown in teleost fish. A gene highly homologous to *hAdo* in the transcriptome of mandarin fish was bioinformatics analyzed in this study, and this *sc*ADO had the molecular basis for achieving the function of thiol dioxygenase: three histidine residues coordinated with Fe(II), PCO/ADO domain, and a “jelly roll” β-barrel structure containing Fe(II) that binds oxygen molecule and substrate. The *sc*ADO clustered with various species ADO in the phylogenetic tree. Furthermore, the expression patterns of *scAdo* were analyzed. *scAdo* was highly expressed in the liver and kidneys, which are the immune-related tissues of mandarin fish. Based on this, it is speculated that *sc*ADO might be involved in the immune process. Hypoxia could also induce an upregulation of *scAdo* expression, which was consistent with the expression characteristics of *Pcos* in plant [34], suggesting that *scAdo* was also a hypoxia-responsive gene. In addition, the IFA result showed that *sc*ADO was located in the cytoplasm, which was consistent with the thiol dioxygenase function of ADO.

BY considering exploring the function of *sc*ADO, this study first demonstrated that *sc*ADO and *h*ADO had a similar function in regulating the stability of *h*RGS4. Therefore, a series of experiments were designed to verify gradually that *sc*ADO also regulated the stability of *sc*RGS4 through the Cys branch of the Arg/N-degron pathway, thus confirming that *sc*ADO was an oxygen-sensing enzyme in mandarin fish. In addition, it was observed that *sc*ADO also degraded during the degradation of *h*RGS4, but the specific mechanism need to be explored by further research. It might be that the two proteins degraded together after their interaction, or *sc*ADO itself had a degradation mechanism. Notably, under the condition of 1% O_2_, the overexpression of *sc*ADO still led to the degradation of *sc*RGS4 despite the inhibited *sc*ADO activity. In recent years, studies have proposed that the overexpression of *h*ADO could inhibit the hypoxic stability of *h*RGS4 [8], which was consistent with the results of this study. The characteristic of *sc*ADO lies in its capability to provide additional possibilities for its function due to its maintenance of certain activities at extremely low oxygen concentrations. If prolyl hydroxylases (PHDs) lose activity under moderate hypoxia, then *sc*ADO can induce the intracellular hypoxic response and rapidly catalyze the deactivation of hypoxic response when oxygen concentration increases. In addition, the HIF signaling pathway based on PHDs responds to hypoxia at the transcriptional level, while the Arg/N-degron pathway Cys branch based on ADO responds to hypoxia at the protein level. The acting mechanisms and response rates are different and are likely to play roles on different levels under various conditions, jointly guiding the physiological activities of the body in response to hypoxia. Therefore, exploring the functions and potential targets of *sc*ADO is of considerable importance for a deep understanding of various hypoxia response mechanisms and their synergistic effects in the body.

As a new oxygen-sensing mechanism in mandarin fish, the Cys branch of the Arg/N-degron pathway based on *sc*ADO not only participates in the hypoxia response, but also affects the virus infection to host, similar to the HIF signal pathway [35]. This study found that *sc*ADO could respond to ISKNV, MRV, and SCRV infections at the expression level and significantly promoted SCRV replication. Subsequently, the treatment of 2,2-DIP demonstrated that the promoting effect of *sc*ADO was also achieved through its thiol dioxygenase activity. Therefore, combined with the oxygen-sensing function of *sc*ADO, a certain target protein of *sc*ADO might have antiviral function, and the specific degradation of *sc*ADO against antiviral protein led to the significant increase in SCRV replication in the presence of *sc*ADO. The two viruses that were unaffected by *sc*ADO in this study, namely ISKNV and MRV, were both DNA viruses, while the SCRV was an RNA virus. This finding might be due to the different mechanisms of translation, replication, assembly, and other processes of DNA and RNA virus in cells, while the antiviral protein regulated by *sc*ADO could only specifically inhibit the infection process of RNA virus and could not affect DNA virus. However, the accuracy of this conjecture can be proven through a comprehensive research on the relationship between *sc*ADO and additional viruses as well as the mechanism of *sc*ADO affecting virus infection. SCRV will replicate extensively in the liver after infecting mandarin fish, leading to pathological changes [21]. Meanwhile, *sc*ADO has the highest expression abundance in the liver of mandarin fish, which is speculated to be related to the function of *sc*ADO to promote SCRV replication. Based on the results of this study, it can be inferred that there may also be a similar relationship between *h*ADO and human viruses, but there is currently no report, subject to further discussion and research. Therefore, exploring the target protein of *sc*ADO and its function in mandarin fish, which is of considerable importance for further understanding the oxygen-sensing mechanisms of mandarin fish and its relationship with virus outbreak, is particularly important.

## 5. Conclusions

The *sc*ADO could lead to the degradation of the RGS4 protein through the Cys branch of the Arg/N-degron pathway. This observation suggested that *sc*ADO was an oxygen-sensing protein, and that an ADO-dependent oxygen-sensing mechanism was present in teleost fish. Furthermore, *sc*ADO could significantly promote the replication of SCRV, which was associated with its activity of thiol dioxygenase. Our work is of considerable importance for further understanding the oxygen-sensing mechanisms of mandarin fish and its relationship with virus outbreak.

## Figures and Tables

**Table 1:** Primers used for cDNA cloning.

Name	Sequences (5′-3′)
*scAdo*-F-EcoR I	GGAATTCGGATGCCAGGTGACAGAAACATG
*scAdo*-R-Kpn I	GGGGTACCTCAGAGGCAGACCTCCGGGCC
*hRgs4*-F-Bgl II	GGGAATTCATGTGCTCCAAACTCGTAGAGATG
*hRgs4-C2A*-F-SalI	GCGTCGACATGGCTAAAGGGCTTGCAGGTC
*hRgs4*-R-Kpn I	GGGGTACCGGCACACTGAGGGACCAGGGA
*scRgs4*-F-EcoR I	GGGAATTCATGTGTAAAGGACTTGCAACA
*scRgs4-C2A*-F-EcoR I	GGGAATTCATGGCTAAAGGACTTGCAACA
*scRgs4*-R-Kpn I	CCGGTACCGGCACCGCCAGTTAACGCCTG

**Table 2:** Primers used for RT-qPCR.

Name	Sequences (5′-3′)
*scAdo*-F	GCGTTCATGGACATCCT
*scAdo*-R	CCTCCGCACCAGAAAT
ISKNV-*Mcp*-F	CAATGTAGCACCCGCACTGACC
ISKNV-*Mcp*-R	ACCTCACGCTCCTCACTTGTC
ISKNV-*Orf008R*-F	TGACCTGTGGCCTAGATGATAAC
ISKNV-*Orf008R*-R	AGAGGCAGAGCAGCAGCATGTAGAGT
ISKNV-*Orf101L*-F	AAGCCGAGGACCCCAAGAAGT
ISKNV-*Orf101L*-R	GTCCTGACCGCCCACCAGTAT
MRV-*Mcp*-F	ATCTCGCCACTTATGACAG
MRV-*Mcp*-R	CAAGAGTTGAGCACATAGTC
MRV-*Orf011R*-F	ACGCAAGAAGTTAGAGCATA
MRV-*Orf011R*-R	CCTGGTAGAATAGAGGTGATT
MRV-*Orf055R*-F	ACAGTGGATCTAGTCAACAT
MRV-*Orf055R*-R	GTACGCAGTCACAGTCAG
SCRV-*Matrixprotein*-F	CGGTTGCCATCTCTTATGA
SCRV-*Matrixprotein*-R	CCTCTGCTTCTGCTATCTG
SCRV-*Nucleoprotein*-F	TCGCATCATTCACTGGATT
SCRV-*Nucleoprotein*-R	TGGCAGAGTAAGGAGACA
SCRV-*Phosphoprotein* -F	ACAGCAGAGGTCTCAAGA
SCRV-*Phosphoprotein*-R	ATTAGCATCCGCAGAAGG
*β-actin*-F	CCCTCTGAACCCCAAAGCCA
*β-actin*-R	CAGCCTGGATGGCAACGTACA

## Data Availability

All data are either provided in the article or are available from the corresponding author upon request.
